# The E3 ubiquitin ligase RNF185 facilitates the cGAS-mediated innate immune response

**DOI:** 10.1371/journal.ppat.1006264

**Published:** 2017-03-08

**Authors:** Qiang Wang, Liyuan Huang, Ze Hong, Zhongshi Lv, Zhaomin Mao, Yijun Tang, Xiufang Kong, Senlin Li, Ye Cui, Heng Liu, Lele Zhang, Xiaojie Zhang, Lindi Jiang, Chen Wang, Qin Zhou

**Affiliations:** 1 Division of Molecular Nephrology and the Creative Training Center for Undergraduates, the Ministry of Education Key Laboratory of Laboratory Medical Diagnostics, the School of Laboratory Medicine, Chongqing Medical University, Chongqing, China; 2 State Key Laboratory of Cell Biology, Institute of Biochemistry and Cell Biology, Shanghai Institutes for Biological Sciences, Chinese Academy of Sciences, Shanghai, China; 3 School of Life Science and Technology, China Pharmaceutical University, Jiangning District, Nanjing, China; 4 Department of Rheumatology, Zhongshan Hospital, Fudan University, Shanghai, China; University of Southern California, UNITED STATES

## Abstract

The cyclic GMP-AMP synthase (cGAS), upon cytosolic DNA stimulation, catalyzes the formation of the second messenger 2′3′-cGAMP, which then binds to stimulator of interferon genes (STING) and activates downstream signaling. It remains to be elucidated how the cGAS enzymatic activity is modulated dynamically. Here, we reported that the ER ubiquitin ligase RNF185 interacted with cGAS during HSV-1 infection. Ectopic-expression or knockdown of RNF185 respectively enhanced or impaired the IRF3-responsive gene expression. Mechanistically, RNF185 specifically catalyzed the K27-linked poly-ubiquitination of cGAS, which promoted its enzymatic activity. Additionally, Systemic Lupus Erythematosus (SLE) patients displayed elevated expression of RNF185 mRNA. Collectively, this study uncovers RNF185 as the first E3 ubiquitin ligase of cGAS, shedding light on the regulation of cGAS activity in innate immune responses.

## Author summary

Ubiquitination has been demonstrated to serve as an effective means to catalyze the rapid, dynamic and versatile regulatory processes that are activated when hosts face microbes. Given the critical functions of cytosolic DNA sensing pathway in anti-viral innate immune responses and the pathogenesis of autoimmune diseases, they are subjected to manifold spatial and temporal modulations shaping the strength and duration of the signaling pathways. Recent progress has characterized the cyclic GMP-AMP synthase (cGAS) as the primary DNA sensor that initiates stimulator of interferon genes (STING)-dependent signaling pathway. However, it remains poorly understood how cGAS activity is modulated dynamically. In this study, we identify E3 ubiquitin ligase RNF185 as a positive regulator of cGAS-STING signaling. Knockdown of RNF185 significantly attenuates IRF3-responsive gene expression. ER-resident RNF185 interacts with cGAS and catalyzes the K27-linked poly-ubiquitination of cGAS upon HSV-1 challenges, which thus potentiates cGAS enzymatic activity. Notably, systemic lupus erythematosus (SLE) patients have elevated expression of RNF185 mRNA. Our study uncovers RNF185 as the first E3 ubiquitin ligase of cGAS and suggests RNF185 as an important target for modulating antiviral response.

## Introduction

The innate immune system serves as the first line of host defense against invading microbes. Upon recognition by an array of host germline-encoded pattern recognition receptors (PRRs), including Toll-like receptors (TLRs), RIG-I-like receptors (RLRs) and DNA sensors, microbial nucleic acids trigger the initiation of intracellular signaling cascades that lead to the induction of type I interferons as well as pro-inflammatory cytokines, which are a prerequisite for eliciting immediate antiviral responses and adaptive immunity to ultimately eradicate the infection [1,2,3,4].

Microbial RNA-sensing machinery and the corresponding downstream signaling cascade have been well characterized during the past decade, whereas the microbial DNA sensing represents a fast evolving field for understanding the corresponding innate immune signaling pathways [5,6,7,8,9]. Various studies have identified several proteins, including Mre11, DAI, RNA polymerase III, IFI16, DDX41 as the potential DNA sensors [10,11,12,13,14]. However, these proteins are not universally essential for detecting microbial DNAs in distinct cell types or *in vivo* [6]. Recently, cyclic GMP-AMP synthase (cGAS) is characterized as a sequence-independent DNA sensor by classical biochemical fractionation strategies coupled with quantitative mass spectrometry [15]. Analyses of cGAS knockout mice reveal its essential roles in fibroblasts, macrophages, and dendritic cells in response to various DNA stimuli transfections and DNA pathogens (DNA viruses, retroviruses, *Listeria monocytogenes* and *Mycobacterium tuberculosis*) infection [16,17,18,19,20]. In addition, cGas^-/-^ mice are more vulnerable to lethal infection after exposure to herpes simplex virus 1 (HSV-1) than wild-type mice [16].

Notably, cGAS possesses nucleotidyl transferase activity, converting ATP and GTP into noncanonical cyclic dinucleotide 2′3′-cGAMP in the presence of DNA [21,22]. As a second messenger, cGAMP directly binds to and activates ER-resident stimulator of interferon genes (STING) [23,24,25]. STING traffics from ER, through the Golgi apparatus, and to the perinuclear microsomes or punctuate structures [25]. During the trafficking processes, the K27-linked poly-ubiquitin chain anchored on STING by AMFR-INSIG1 complex recruits the TANK-binding kinase 1 (TBK1), which causes STING and TBK1 to congregate simultaneously in the same compartment [26]. Importantly, the DNA-triggered assembly of STING-TBK1 complex is critical for TBK1 activation, followed by activating the transcriptional factor IRF3, thus inducing expression of type I interferons and pro-inflammatory cytokines.

Protein post-translational modifications, such as phosphorylation, ubiquitination, and SUMOylation, are central to the host innate immune regulations [27,28]. cGAS is potentially subjected to a couple of modifications [29,30,31]. For example, the glutamylases TTLL6 catalyzes poly-glutamylation of cGAS and impedes its DNA-binding activity, whereas TTLL4-mediated mono-glutamylation of cGAS blocks its synthase activity. The carboxypeptidases CCP6 and CCP5 reverse the above processes respectively, thus promoting the cGAS activation [29]. The protein kinase Akt phosphorylates cGAS and suppresses its enzymatic activity [30]. However, it remains unknown whether cGAS is modulated by ubiquitination. A thorough study on the regulation of cGAS activity is deserved because the aberrant activation of cGAS causes severe autoimmune or autoinflammatory disorders, such as systemic lupus erythematosus (SLE) and Aicardi Goutières syndrome (AGS) [32,33,34].

The E3 ubiquitin ligase RNF185 potentially modulated the osteogenesis or protein quality control on the ER [35,36,37]. In this study, we characterized the ER-resident RNF185 as a positive regulator of the cGAS-STING signaling. RNF185 interacted with cGAS and catalyzed the K27-linked poly-ubiquitination of cGAS upon HSV-1 challenges, which markedly potentiated the enzymatic activity of cGAS. Additionally, SLE patients exhibited elevated RNF185 mRNA expression.

## Results

### Identification of RNF185 as a new regulator of cytosolic DNA sensing pathway

**Fig 1:**
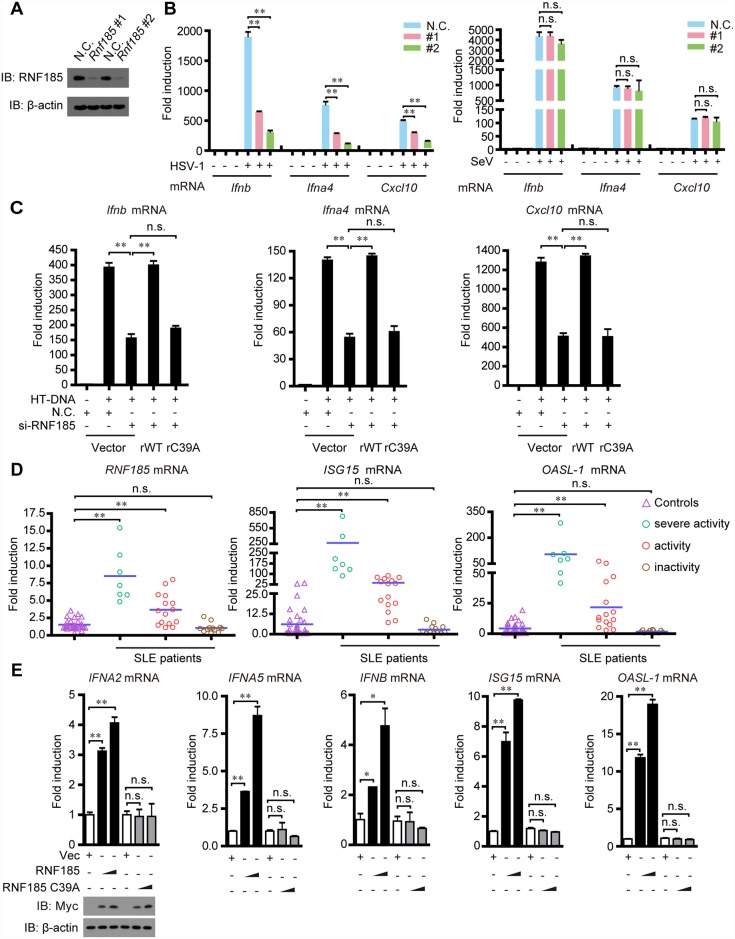
Identification of RNF185 as a new regulator in cytosolic DNA sensing pathway. (A) L929 cells were transfected with the negative control (N.C.) or *Rnf185* siRNAs. Cell lysates were immunoblotted with the indicated antibodies. (B) The indicated siRNAs were transfected into L929 cells. Induction of *Ifnb*, *Ifna4* and *Cxcl10* mRNAs was measured by quantitative PCR after HSV-1 (MOI = 2) invasion (left panel) or SeV infection (right panel) for 6h. (C) L929 cells were transfected with the negative control (N.C.) or RNF185 siRNA and then rescued with the indicated siRNA-resistant RNF185 constructs. After HT-DNA stimulation, induction of *Ifnb*, *Ifna4* and *Cxcl10* mRNAs was measured by quantitative PCR. (D) Comparisons of RNF185 gene expression and type I IFN-inducible gene (*OASL-1* and *ISG15*) expression in PBMCs from patients with SLE (divided into three groups based on the systemic lupus erythematosus disease activity index (SLEDAI) score: 2 ≤ score ≤ 6, inactivity; 7 ≤ score ≤ 14, activity; 15≤ score ≤ 38, severe activity), and 32 healthy donors. P< 0.0001 (Mann-Whitney test). Each symbol represents an individual subject, and horizontal lines show the mean. (E) PBMCs were transfected with empty vector, an expression vector for Myc-RNF185 (0.6 and 0.9μg, wedge) or Myc-RNF185 C39A (0.6 and 0.9μg, wedge). 32h after transfection, Induction of *IFNA2*, *IFNA5*, *IFNB*, *ISG15* and *OASL-1* mRNAs was measured by quantitative PCR. The cell lysates were immunoblotted with indicated antibodies. β-actin was served as loading control. Data from B, C and E are presented as means ± S.D. from three independent experiments. *, *P* < 0.05; **, *P* < 0.01. n.s., not significant.

Because polyubiquitination has emerged as an important regulatory mechanism for cGAS-STING signaling [27,28], we speculated whether additional E3 ubiquitin ligases catalyze the ubiquitination of key signaling molecules and thereby regulate innate antiviral response. We noticed that RNF185 contains a RING domain, a signature of ubiquitin E3 ligases, and shares a high degree of sequence identity (approximate to 70%) with RNF5, which catalyzed the ubiquitin-mediated degradation of STING (S1A Fig). To explore the potential role of RNF185, we screened out the specific and effective siRNAs (mouse *Rnf185* siRNA 1# and mouse *Rnf185* siRNA 2#) (Fig 1A). As expected, silencing of *Rnf185* markedly attenuated the expression of the IRF3-responsive genes (*Ifnb*, *Ifna4* and *Cxcl10*) in L929 cells, stimulated by the herring testis DNA (HT-DNA) transfection (S2A Fig) or the DNA virus HSV-1 infection (Fig 1B, left panel). In contrast, the abundance of *Ifnb*, *Ifna4* or *Cxcl10* mRNAs induced by RNA mimic poly(I:C) transfection (S2A Fig) or RNA virus Sendai virus (SeV) infection (Fig 1B, right panel) was comparable between *Rnf185* knockdown and wild-type L929 cells.

Similarly, knockdown of *Rnf185* in Raw264.7 cells also significantly attenuated the expression of the IRF3-responsive genes (*Ifnb*, *Ifna4* and *Cxcl10*), when challenging cells with HSV-1 (S3A Fig). In contrast, the induction of the IRF3-responsive genes was marginally affected in *Rnf185* knockdown Raw264.7 cells when challenging cells with SeV (S3B Fig). To make it more physiologically relevant, we next probed the role of RNF185 in primary cells. We confirmed RNF185 expression was also efficiently reduced in the BMDMs (bone marrow derived macrophages) transfected with the indicated siRNAs (S3C Fig). Consistently, silencing of *Rnf185* markedly attenuated the expression of the IRF3-responsive genes (*Ifnb*, *Ifna4* and *Cxcl10*) in BMDMs stimulated by HSV-1 (S3D Fig). In contrast, the abundance of *Ifnb*, *Ifna4* or *Cxcl10* mRNAs induced by SeV infection was comparable between *Rnf185* knockdown and wild-type BMDMs (S3E Fig). Furthermore, RNF185 knockdown in BMDMs resulted in obvious increase in HSV-1 titer as compared with controls by standard plaque assay (S3F Fig, left panel). However, RNF185 knockdown did not influence Sendai virus replication as checked by qPCR analysis (S3F Fig, right panel). These data suggest that RNF185 specifically regulates cytosolic DNA sensing pathway.

To rule out potential off-target effects of the RNF185 siRNA, we generated two RNA interference (RNAi)-resistant RNF185 constructs, named rRNF185 WT and rRNF185 C39A, in which silent mutations were introduced into the sequence targeted by the siRNA without changing the amino acid sequence of the corresponding proteins. L929 cells were first transfected with control or RNF185 siRNA followed by introduction of control or indicated rRNF185 plasmids, respectively. Then the induction of IRF3-responsive genes (*Ifnb*, *Ifna4* and *Cxcl10*) was measured after HT-DNA stimulation. As shown in Fig 1C, the induction of *Ifnb*, *Ifna4* and *Cxcl10* was restored by rRNF185 WT, but not rescued by rRNF185 C39A. These data suggest that RNF185 potentially modulates the cytosolic DNA sensing pathway depending on its enzymatic activity.

The immune sensing of microbial DNA is critical for triggering immediate immune responses and the subsequent adaptive immunity [3]. However, inappropriate provocation of the immune system by aberrant self-DNA, which should be cleared under normal conditions, contributes to the pathogenesis of certain autoimmune diseases, such as systemic lupus erythematosus (SLE) [38,39]. Since RNF185 might be involved in regulating cGAS-mediated DNA sensing pathway, we further examined the mRNA expression levels of *RNF185* as well as *ISG15* and *OASL-1* (type I IFNs inducible genes) in peripheral blood mononuclear cells (PBMCs) isolated from SLE patients and healthy controls by QPCR analysis. The *RNF185* mRNA expression was significantly up-regulated in SLE patients as compared with healthy controls (Fig 1D). The *ISG15* and *OASL-1* mRNA expression were also increased in SLE patients as compared with healthy controls (Fig 1D). Interestingly, ectopic-expression of wild-type RNF185 in PBMCs potentiated the expression of *ISG15* and *OASL-1* mRNA as well as *IFNA2*, *IFNA5* and *IFNB* mRNA, whereas the mutant RNF185 C39A could not (Fig 1E).

In addition, we treated PBMCs with purified IFNα2a in different doses, and observed that IFNα2a could efficiently induce the expression of *ISG15* and *OASL-1* mRNA in early and late time points (S2B and S2C Fig). In contrast, the *RNF185* mRNA expression was barely affected at the early and late phase of IFNα2a treatment (S2B and S2C Fig). To make the experiment more physiologically relevant, we stimulated the PBMCs with different titrations of serum isolated from SLE patients and healthy controls. As expected, the cells treated with the serum from SLE patients produced much more *ISG15* and *OASL-1* mRNA than did those from healthy controls (S2D and S2E Fig). Notably, serum from SLE patients displayed no substantial effect on the expression of RNF185 mRNA as compared with those from healthy controls in early and late time points (S2D and S2E Fig). These data suggest that no positive feedback loop exists between *RNF185* mRNA expression and Interferons production.

### Silencing of *Rnf185* attenuates cytosolic DNA-triggered IRF3 activation

**Fig 2:**
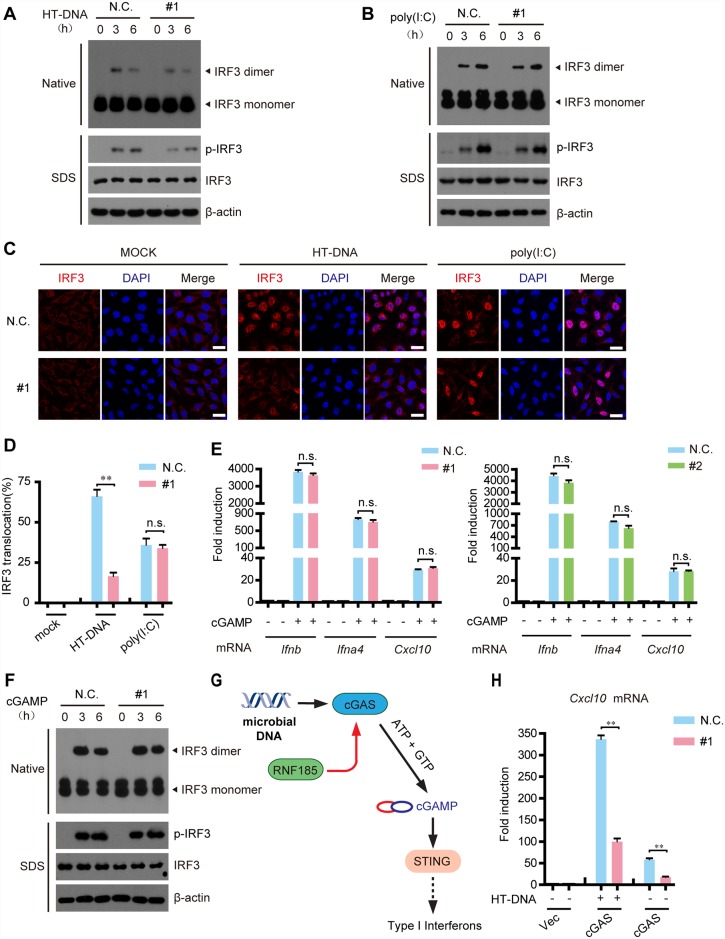
Silencing of RNF185 attenuates exogenous DNA-induced IRF3 activation. (A and B) The indicated siRNAs were transfected into L929 cells. Forty-eight hours after transfection, cells were treated with HT-DNA (A) or poly(I:C) (B) for the indicated time periods, and cell extracts were analyzed for IRF3 dimerization and IRF3 phosphorylation by native PAGE and SDS-PAGE, respectively. (C) Wild-type or *Rnf185* knockdown L929 cells treated with HT-DNA or poly(I:C) for 4h, were stained with the antibody against IRF3, and imaged by confocal microscopy. Scale bars, 50μm. (D) Cells with nuclear IRF3 staining are counted as a percentage of total cells (n = 100 cells per sample). (E) Digitonin permeabilized wild-type or *Rnf185* knockdown L929 cells were incubated with or without cGAMP for 30 min at 37°C, and then the media was replaced. Six hours after cGAMP delivery, induction of *Ifnb*, *Ifna4* and *Cxcl10* mRNAs was measured by quantitative PCR. (F) The indicated siRNAs were transfected into L929 cells. Forty-eight hours after transfection, cells were incubated with or without cGAMP for 30 min at 37°C, and then the media was replaced. After cGAMP delivery for the indicated time periods, cell extracts were analyzed for IRF3 dimerization and IRF3 phosphorylation by native PAGE and SDS-PAGE, respectively. (G) Schematic diagram of the signaling node targeted by RNF185. (H) L929/cGAS cells were transfected with the negative control (N.C.) or *Rnf185* siRNA. 48h after transfection, cells were treated with or without HT-DNA. Induction of *Cxcl10* mRNA was measured by quantitative PCR. Data from D, E and H are presented as means ± S.D. from three independent experiments. **, P < 0.01. n.s., not significant.

The dimerization and phosphorylation of IRF3 as well as the phosphorylation of TBK1 are hallmarks of the cytosolic DNA-triggered signaling. These processes were apparently inhibited in *Rnf185* knockdown L929 cells, when stimulating cells with HT-DNA (Figs 2A and S4A). However, poly(I:C)-induced dimerization or phosphorylation of IRF3 as well as the phosphorylation of TBK1 were barely affected when silencing *Rnf185* (Figs 2B and S4B). In addition, the nuclear translocation of IRF3 triggered by HT-DNA was markedly crippled when knocking down *Rnf185* in L929 cells (Fig 2C and 2D), whereas the nuclear translocation of IRF3 triggered by poly(I:C) remained intact in *Rnf185* knockdown L929 cells (Fig 2C and 2D). Collectively, these data indicate that RNF185 is essential for the cytosolic DNA-induced IRF3 activation.

Interestingly, silencing of RNF185 apparently did not affect the expression of IRF3-responsive genes (*Ifnb*, *Ifna4* and *Cxcl10*) induced by cGAMP (Fig 2E). Consistently, cGAMP-triggered dimerization and phosphorylation of IRF3 were barely affected when silencing *Rnf185* (Fig 2F). Therefore, we reasoned that RNF185 played a role on the upstream of STING (Fig 2G). To substantiate, silencing of *Rnf185* markedly attenuated the induction of *Cxcl10* and *Ifnb* as well as the phosphorylation of TBK1 and IRF3 by cGAS in L929 cells, stimulated with or without HT-DNA. (Figs 2H, S4C and S4D), which suggest that RNF185 may modulate the cGAS signalsome.

### RNF185 specifically interacts with cGAS

**Fig 3:**
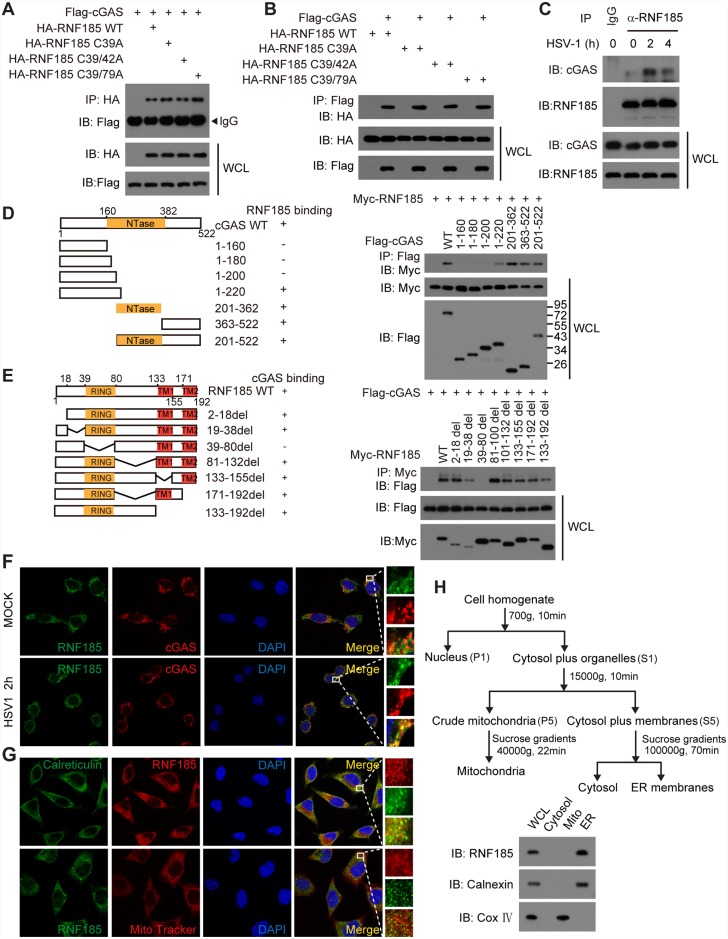
RNF185 interacts with cGAS. (A and B) HEK293T cells were transfected with the indicated plasmids. Twenty-four hours after transfection, cell lysates were immunoprecipitated with an anti-HA antibody (A) or an anti-Flag antibody (B) and then immunoblotted with the indicated antibodies. (C) After mock or HSV-1 (MOI = 10) stimulation for the indicated time periods, lysates from BMDMs were immunoprecipitated with an anti-RNF185 antibody or normal IgG, and then immunoblotted with the indicated antibodies. (D) Schematic diagram of cGAS and its truncation mutants (left panel). Flag-tagged cGAS or its mutants were individually transfected into HEK293T cells along with Myc-tagged RNF185. The cell lysates were immunoprecipitated with an anti-Flag antibody and then immunoblotted with the indicated antibodies (right panel). (E) Schematic diagram of RNF185 and its truncation mutants (left panel). Myc-tagged RNF185 or its mutants were individually transfected into HEK293T cells along with Flag-tagged cGAS. The cell lysates were immunoprecipitated with an anti-Myc antibody and then immunoblotted with the indicated antibodies (right panel). (F) L929 cells were left untreated or infected with HSV-1 (MOI = 1) for 2hr, and then were stained with indicated antibodies and imaged by confocal microscopy. Scale bars, 25μm. (G) L929 cells were stained with indicated antibodies and imaged by confocal microscopy. The mitochondria were stained with MitoTracker. Scale bars, 25μm. (H) Immunoblot analysis of fractionated L929 cells. Control antibodies indicated accuracy of fractionation (Calnexin, ER; COX IV, mitochondria).

To address the association between RNF185 and cGAS, HA-tagged RNF185 and Flag-tagged cGAS were transfected individually or together into HEK293T cells, followed by coimmunoprecipitation (coIP) assays. As expected, HA-tagged RNF185 associated with Flag-tagged cGAS (Fig 3A and 3B). It was predicted that the cysteines in the RING domain of RNF185 are critical for its catalytic activity. Several RNF185 mutants were therefore generated, including RNF185 C39A (Cys to Ala mutation at 39 residues), RNF185 C39/42A (Cys to Ala mutation at both 39 and 42 residues) and RNF185 C39/79A (Cys to Ala mutation at both 39 and 79 residues), all of which were deprived of the potential E3 ubiquitin ligase activity (see below). It was observed that cGAS associated with these RNF185 mutants as well as with wild-type RNF185 (Fig 3A and 3B), indicating that the E3 ubiquitin ligase activity of RNF185 was dispensable for its association with cGAS. We further confirmed the weak endogenous association between cGAS and RNF185 (Fig 3C). Notably, the endogenous association between cGAS and RNF185 was substantially enhanced upon HSV-1 infection (Fig 3C).

A series of deletion mutants of cGAS and RNF185 were employed to map the domains responsible for RNF185-cGAS interaction (Fig 3D and 3E, left panel). The C-terminal domain of cGAS (amino acids 201–522) and the RING domain of RNF185 (amino acids 39–80) were required for the interaction (Fig 3D and 3E, right panel).

Confocal microscope imaging revealed that endogenous RNF185 partially co-localized with endogenous cGAS in resting cells (Figs 3F and S5A), and this co-localization was enhanced after HSV-1 infection (Figs 3F and S5A). Confocal microscopy and subcellular fractionation analysis confirmed that RNF185 were predominantly expressed on ER membrane, but not on mitochondria membrane (Fig 3G and 3H). Taken together, these data indicate that RNF185 is a novel member of the cGAS signalsome *in vivo*.

### RNF185 catalyzes the K27-linked poly-ubiquitination of cGAS

**Fig 4:**
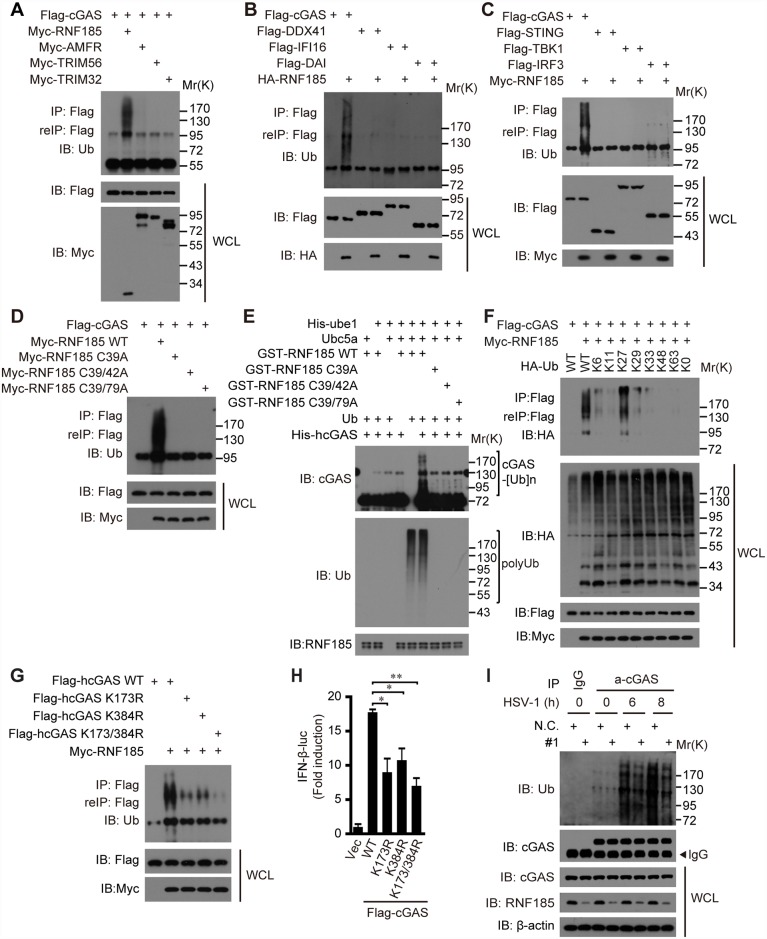
RNF185 catalyzes K27-linked poly-ubiquitination of cGAS. (A, B and C) HEK293T cells were transfected with the indicated plasmids. Thirty-two hours after transfection, cell lysates were immunoprecipitated with an anti-Flag antibody. The immunoprecipitates were denatured and re-immunoprecipitated with an anti-Flag antibody, and then analyzed by immunoblotting with the indicated antibodies. (D) Myc-tagged RNF185 or its mutants were individually transfected into HEK293T cells along with Flag-tagged cGAS, cell lysates were subjected to a two-step immunoprecipitation and then immunoblotted with the indicated antibodies. (E) The ubiquitination reaction mixture contains E1, E2, Ub, cGAS and RNF185 or RNF185 mutants as indicated. After incubation for 1 hr, the mixture was detected by immunoblotting with cGAS-specific or Ub-specific antibodies. (F) HEK293T cells were transfected with Flag-tagged cGAS and Myc-tagged RNF185 along with Ub or its mutants. Cell lysates were subjected to a two-step immunoprecipitation, and then immunoblotted with the indicated antibodies. (G) Flag-tagged cGAS or its mutants were individually transfected into HEK293T cells along with Myc-tagged RNF185. Cell lysates were subjected to a two-step immunoprecipitation, and then immunoblotted with the indicated antibodies. (H) HEK293T/STING cells were transfected with the cGAS or its mutants along with the IFN-β promoter reporter and pTK-Renilla reporter plasmids for thirty-two hours before luciferase assays were performed. Data are presented as means ± S.D. from three independent experiments. **, P < 0.01. (I) L929 cells transfected with the negative control (N.C.) or *Rnf185* siRNA were infected with or without HSV-1 (MOI = 10) for the indicated time periods, and the lysates were subjected to denaturing immunoprecipitation with an anti-cGAS antibody or normal IgG, and then analyzed by immunoblotting with the indicated antibodies.

As an E3 ubiquitin ligase, RNF185 could catalyze the ubiquitin-mediated degradation BNIP1, CFTR and Dvl2 [35,36,37]. Our i*n vitro* ubiquitination assays confirmed that RNF185 could catalyze the formation of poly-ubiquitin chains, whereas RNF185 C39A, RNF185 C39/42A or RNF185 C39/79A could not (Fig 4E).

Our above data uncovered the importance of the E3 ubiquitin ligase activity of RNF185 for the cytosolic DNA sensing pathway (Fig 1C). Therefore, we wondered whether the cGAS was the authentic substrate of RNF185. To explore this possibility, Flag-tagged cGAS was co-transfected respectively with RNF185 or other E3 ligases known in the STING pathway. The cell lysates were subjected to immunoprecipitation with anti-Flag, and then the immunoprecipitates were denatured, followed by re-immunoprecipitation again with anti-Flag; the precipitates were finally analyzed by immunoblotting with anti-ubiquitin. Notably, cGAS was markedly poly-ubiquitinated in the presence of RNF185 (Fig 4A). In contrast, other E3 ligases could not catalyze the ubiquitination of cGAS (Fig 4A). Apparently, RNF185 could not catalyze the polyubiquitination of other potential DNA sensors [11,13,14] (Fig 4B), neither could it catalyze the polyubiquitination of STING, TBK1 or IRF3 (Fig 4C). In addition, the catalytically inactive mutants RNF185 C39A, RNF185 C39/42A or RNF185 C39/79A failed to catalyze the polyubiquitination of cGAS inside cells (Fig 4D). *In vitro* ubiquitination assay further confirmed that the wild-type RNF185 catalyzed the formation of poly-ubiquitin chains on cGAS, whereas the RNF185 C39A, RNF185 C39/42A or RNF185 C39/79A could not (Fig 4E). Thus, cGAS is a new substrate of the RNF185.

A panel of ubiquitin mutants, including those containing a point mutation at a corresponding lysine and those with all lysines mutated to arginines except for the indicated one, were employed to dissect the polyubiquitin chain linkage on cGAS. As expected, RNF185 catalyzed the poly-ubiquitination of cGAS in the presence of wild-type ubiquitin, whereas the poly-ubiquitination of cGAS was completely abolished when using the ubiquitin K0 mutant (Ubiquitin with all lysine residues mutated to arginine). Notably, the modification reappeared when K27, rather than other lysines, was reintroduced into the ubiquitin K0 mutant (Fig 4F). Moreover, cGAS was not poly-ubiquitinated when using ubiquitin K27R (S5B Fig), whereas cGAS was polyubiquitinated as well by K6R, K11R, K29R, K33R, K48R and K63R (S5B Fig). Collectively, the data indicate that RNF185 catalyzes the formation of the K27-linked polyubiquitin chains on cGAS.

To identify the potential poly-ubiquitination sites on cGAS, we carried out a systematic lysine (K) to arginine (R) mutation scanning. When the two lysines (K173 and 384) on cGAS were all mutated to arginines, the poly-ubiquitination of cGAS was almost completely abolished (Fig 4G). To substantiate, cGAS mutants could induce the IFN-β-luciferase reporter gene to a much lower level than their wild-type one (Fig 4H). In addition, the expression of *Cxcl10* and *Ifnb* mRNAs as well as the phosphorylation of TBK1 and IRF3 triggered by cGAS K173R/384R mutant were much lower than by wild-type cGAS in L929 cells stimulated with or without HT-DNA (S5C and S5D Fig).

There was some background poly-ubiquitination of the endogenous cGAS in resting cells (Fig 4I). The endogenous cGAS was robustly poly-ubiquitinated upon HSV-1 infection. Importantly, the poly-ubiquitination of cGAS was markedly reduced in *Rnf185* knockdown L929 cells (Fig 4I). Taken together, these data establish that cGAS is an authentic substrate of RNF185, which catalyzes the K27-linked poly-ubiquitination of cGAS.

### The ubiquitination of cGAS potentiates its catalytic activity

**Fig 5:**
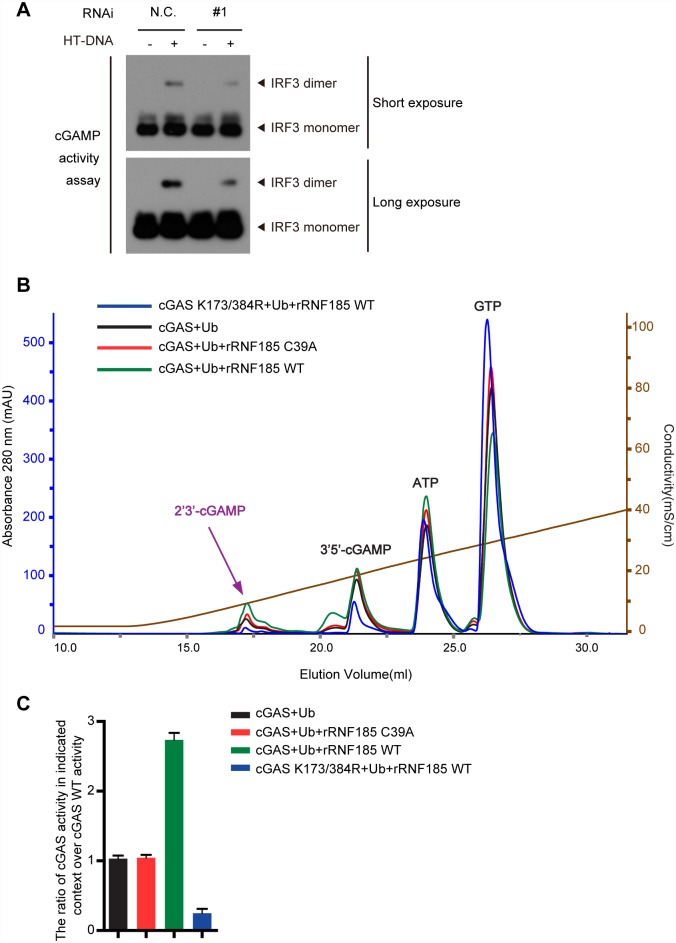
The ubiquination of cGAS potentiates its activation. (A) L929 cells transfected with the negative control (N.C.) or *Rnf185* siRNA were stimulated with HT-DNA for 6 hr, and extracts from these cells were used to prepare heat-resistant supernatants, which were delivered to permeabilized fresh L929 cells to stimulate IRF3 dimerization. (B) The reaction products from cGAS (10 μM) or cGAS mutants (10 μM) as well as ubiquitinated cGAS (10 μM) catalyzed by recombinant RNF185 WT (0.2 μM) or RNF185 C39A (0.2 μM) were analyzed by ion exchange chromatography. Salmon sperm DNA at 0.2 mg/ml was used to stimulate cGAS catalytic activity. (C) Specific activity of recombinant cGAS was quantified by the ratio of the yield of 2′3′-cGAMP to the total amount of ATP plus GTP, and the yield of 2′3′-cGAMP was calculated by area in evaluated peaks under indicated curve in (B) using UNICORN version 5.20 software (GE Healthcare). The value for the activity of wild type cGAS is set as 1.0. Data are presented as means ± S.D. from three independent experiments.

To probe the functional role of the ubiquitination of cGAS, L929 cells were transfected with RNF185 siRNA and stimulated with HT-DNA. cGAMP levels in the infected cell lysates were indirectly measured through incubation of PFO-permeabilized fresh L929 cells with those lysates, and the IRF3 dimerization was checked. As expected, knocking down RNF185 in L929 cells resulted in a significant reduction of cGAMP production upon HT-DNA transfection (Fig 5A), which indicate that RNF185 is required for activating cGAS in response to cytosolic DNA challenge.

The *in vitro* enzymatic activity assay was further employed to assess the effect of cGAS ubiquitination on its cGAMP synthetic activity. Briefly, the purified recombinant proteins of cGAS or cGAS mutants from bacteria were subjected to the *in vitro* ubiquitination reaction, and then they were incubated with the salmon sperm DNA in the presence of ATP and GTP. The production of cGAMP was analyzed by ion exchange chromatography. As expected, recombinant RNF185 (rRNF185) promoted recombinant cGAS to produce much more 2′3′-cGAMP than the rRNF185 C39A mutant (Fig 5B and 5C). In addition, the polyubiquitin-chain-deficient cGAS mutant could marginally synthesize 2’3’-cGAMP in the presence of rRNF185 (Fig 5B and 5C). Taken together, these data reveal that the poly-ubiquitination of cGAS promotes its catalytic activity.

### RNF185 is important for innate antiviral responses

**Fig 6:**
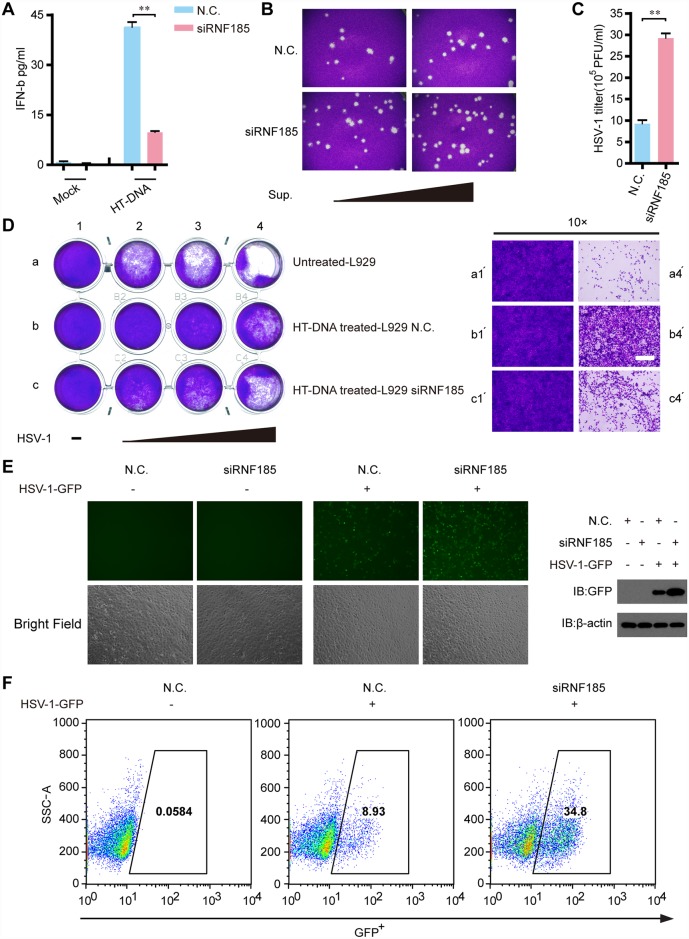
RNF185 modulates anti-viral cGAS-STING signaling. (A) L929 cells were transfected with the negative control (N.C.) or *Rnf185* siRNA. Forty-eight hours after transfection, cells were stimulated with HT-DNA, and the supernatants were collected and assayed for IFN-β production by ELISA. (B and C) L929 cells transfected with the negative control (N.C.) or *Rnf185* siRNA were infected with HSV-1 (MOI = 1) for 36h.The titers of HSV-1 were determined by standard plaque assay. Viral plaques were observed by microscopy (B) and the viral titer was calculated (C). (D) L929 cells transfected with the negative control (N.C.) or *Rnf185* siRNA were stimulated with mock or HT-DNA. Equal volumes of culture supernatants from these treatments were applied to fresh MEF cells, followed by HSV-1 (MOI = 2, 6, and 10, wedge) infection for 48h. The proliferation of cells was examined by crystal violet staining (left panel). The right panel showed the zoom in view of cells proliferation. Scale bars, 100μm. (E) HSV-1-GFP (MOI = 1) replication in MEFs transfected with the negative control (N.C.) or *Rnf185* siRNA after infection for 36h was visualized by fluorescence microscopy (left panel). Immunoblotting analysis of partial cell extracts with the anti-GFP antibodies. β-actin was served as loading control. Data are representative of three independent experiments. (F) GFP-positive cells from E were quantified by flow cytometry. Data from A and C are presented as means ± S.D. from three independent experiments. **, *P* < 0.01.

The robust induction of IFN-β and interferon-stimulated genes (ISGs) represents one of the immediate responses to cytosolic DNA virus infections. ELISA assays indicated that the production of IFN-β was markedly reduced in *Rnf185* knockdown L929 cells stimulated with HT-DNA (Fig 6A). Standard plaque assay revealed that RNF185 knockdown resulted in nearly 3-fold increase in HSV-1 virus titer as compared with controls **(**Fig 6B and 6C). Since IFN-β protects host cells against viruses, we assessed if RNF185 played a role in restricting HSV-1 infection. MEF cells were pretreated respectively with culture supernatants from HT-DNA-stimulated *Rnf185* knockdown L929 cells or wild-type L929 cells, followed by HSV-1 infection. Fresh cells pretreated with culture supernatants from *Rnf185* knockdown L929 cells were more permissive to HSV-1 infection (Fig 6D). We next investigated whether RNF185 modulated virus replication by challenging cells with HSV-1-GFP. It was observed that the cells with *Rnf185* knockdown showed considerably increased numbers of HSV-1-GFP positive cells (Fig 6E and 6F). Taken together, these data indicate that RNF185 is important for innate antiviral responses.

## Discussion

Much is known about the signal transduction triggered by the cytosolic DNAs [24,40]. Unlike other sensors (DAI, IFI16, DDX41, Mre11) [10,11,13,14], cGAS was characterized as a universal sensor that initiates the STING signaling in multiple cell types triggered by many stimuli [15]. Although several recent studies have uncovered key structural features associated with DNA recognition by cGAS as well as the catalytic mechanisms of cGAS generating cGAMP [22,41,42,43,44], it is not well understood how the cGAS activity is modulated dynamically in response to pathogenic or self DNA.

In this study, we performed unbiased RNAi-based screening (S6 Fig), and identified a novel E3 ubiquitin ligase RNF185 to directly modulate cGAS action. Several lines of evidence substantiate the important function of RNF185 in the cytosolic DNA sensing pathway. (a) Knocking down RNF185 specifically attenuated the expression of IRF3-responsive genes induced by DNA mimics transfection or DNA virus HSV-1 infection, but not by RNA mimic transfection or RNA virus SeV infection. (b) The effect produced by RNF185 knockdown was reversed by exogenously expressing a siRNA-resistant rRN185, not by expressing the enzymatic inactive RNF185 mutant, indicating that the regulatory function of RNF185 was dependent on its enzymatical activity. (c) The phosphorylation, dimerization and nuclear translocation of IRF3 triggered by cytoslic DNAs were markedly crippled in RNF185 knockdown cells. (d) Silencing of RNF185 was more permissive to the HSV-1infection, establishing that RNF185 was important for the innate antiviral responses.

In most cases, HSV-1 infection brings about herpetic encephalitis or genital disease in a living host [45,46]. In our data, we noticed that RNF185 knockdown resulted in nearly 3-fold increase in HSV-1 virus titer as compared with controls. However, it is worthwhile to explore in the future clinical study whether a 3-fold statistically significant difference in a HSV titer could potentially affect herpetic encephalitis or genital disease in a living host.

RNF185 was previously shown to catalyze the ubiquitin-mediated degradation of several proteins (BNIP1, CFTR and Dvl2) and modulate the protein quality control on ER [35,36,37]. In this study, we characterized cGAS as a new substrate of RNF185. (a) RNF185 specifically associated with cGAS, and this association was markedly increased upon HSV-1 challenge, indicating that this association was transient and dynamic. (b) Wild-type RNF185, but not its enzymatic inactive mutants, could catalyze the poly-ubiquitination of cGAS, as evidenced by the two-step immunoprecipitation or *in vitro* ubiquitination assay. (c) Site-directed mutagenesis revealed that lysines 173 and 384 on cGAS were major acceptor sites of the polyubiquitin chain. (d) RNF185 specifically catalyzed the K27-linked polyubiquitin chain on cGAS. (e) DNA virus infection induces the ubiquitination of endogenous cGAS by RNF185, as evidenced by the observation that RNAi-mediated silencing of RNF185 diminished these effects. (f) The ubiquitination of cGAS potentiates its enzymatic activity and boosts the production of cGAMP. Taken together, RNF185 is an authentic E3 ubiquitin ligase for cGAS and promotes its activation.

It is recently well established that the aberrant activation of the cGAS-STING signaling by self-DNA causes severe autoimmune or auto-inflammatory disorders, such as SLE [38,39,47]. We found that RNF185 mRNA expression is substantially elevated in PBMCs from SLE patients. Generation of RNF185-deficient mice in the future will further elucidate the functional relevance of RNF185 in SLE.

cGAS-STING signaling is essential for monitoring mitochondrial DNA (mtDNA) released into cytoplasm during mitochondrial membrane permeabilization or stress [48,49,50]. It is also indispensable for sensing damaged DNA leaked into cytoplasm, resulting from ATM (Ataxia-telangiectasia mutated) deficiency or exogenous genotoxic stress [51]. In particular, cGAS-STING signaling is important in sensing and responding to tumor cell-derived DNA [52,53]. Future investigation is expected to uncover the potential roles of RNF185 in mitochondrial stress, DNA damage, and tumor immunity. Insights from these studies might substantiate RNF185 as a potential therapeutic target for further clinical trials.

Ubiquitination is a versatile post-translational modification critical in innate immunity [27,54]. Different linkages of polyubiquitin chains anchored on target proteins produce specific physiological or pathological consequences. K48- and K63-linked polyubiquitin chains have been extensively used in regulating the TLR and RLR signaling pathways [27,54]. Apparently, ubiquitin-mediated modulation of the cGAS-STING signaling is no exception. For example, RNF5 promotes K48-linked poly-ubiquitination of STING, thus dampening the cytosolic virus-triggered immune responses [55]. Additionally, E3 ubiquitin ligases TRIM56 and TRIM32 respectively facilitate the K63-linked poly-ubiquitination of STING and positively regulate the host anti-microbial responses [56,57]. Given the diversity of the STING poly-ubiquitination, it is worthwhile to explore whether cGAS is also modulated by other forms of poly-ubiquitination. It remains to address whether the stability of cGAS is dynamically modulated by the ubiquitin-proteasome system.

## Materials and methods

### Ethics statement

Ethical approval for this study was granted by the Clinical Research Ethics Committee of Zhongshan Hospital, Fudan University School of Medicine. All the participants gave written informed consent before enrollment. Age matched 32 healthy volunteers were recruited as controls. All healthy volunteers used as controls also provided written informed consent.

### Clinical samples

We collected 34 patients all fulfilling the American College of Rheumatology classification criteria for SLE [58]. These patients included two male patients and thirty-two female patients, 18 to 48 years old, averaging 37 years old. All patients were new-onset and not being treated before. Patients who coincided with other autoimmune diseases were excluded. All subjects were screened for infectious conditions.

Peripheral blood (8 ml) was sampled in 10ml EDTA containing Vacutainer K2E (BD biosciences). Peripheral blood mononuclear cells (PBMCs) were separated by density gradient centrifugation using Ficoll-Paque PLUS (GE Healthcare) and the RNA was extracted from PBMCs using TRIzol reagent (Invitrogen) according to the manufacturer’s instructions.

### Cell culture and transfection

L929 cells (ATCC) were cultured in RPMI 1640 medium (Invitrogen) supplemented with 10% FBS and 1% penicillin-streptomycin. HEK293T (ATCC), HEK293FT (kindly provided by Ke Lan’ lab in Shanghai Pasteur Institute), MEFs (ATCC) and RAW264.7 cells (ATCC) were maintained in DMEM plus 10% FBS (Gibco), supplemented with 1% penicillin-streptomycin (Invitrogen). Vero cells (kindly provided by Ke Lan’ lab in Shanghai Pasteur Institute) were cultured in MEM (SAFC Biosciences) supplemented with 10% FBS and 1% penicillin-streptomycin. BMDMs (bone marrow derived macrophages) were prepared as described previously [59]. HEK293T cells and HEK293FT cells were transfected by standard calcium phosphate precipitation method. Other cells were transfected by Lipofectamine 2000 (Invitrogen) according to the manufacturer’s instructions.

### siRNA-based screening

The individual SMART pool siRNA probes (Dharmacon) against a mouse ubiquitin-E3-ligase sub-library of 43 genes encoding RING finger proteins were transfected into L929 cells. 48h after transfection, cells were left uninfected or infected with HSV-1 for 6h, and then cells were directly collected into lysis buffer and cDNA was obtained according to the manufacturer's instruction (Cells-to-cDNA II Kit, Invitrogen). The quantifications of gene transcripts were performed by real-time PCR.

### Construction of stable cell lines

HEK293T/STING cells were originated from HEK293T cells selected by Zeocin ^™^ (Invitrogen, 500ug/ml) following transfection with pCMV-Zeo-STING [60]. L929/cGAS cells and L929/cGAS K173/384R cells were established by transducing the phageflag-cGAS or phageflag-cGAS K173/384R lentiviruses into L929 cells followed by sorting with flow cytometry. Lentiviruses production was performed according to the manufacturer’s instructions. Briefly, HEK293FT cells plated on 100-mm dishes were transfected with the indicated lentiviral expression plasmid (15ug) together with the PSPA (10ug) and the PMD2G (5ug). The viral particles were collected at 48h, filtered by 0.45um membrane filter and used to infect the indicated cells in the presence of polybrene (4ug/ml). After transfection for 48h, the positive cells were sorted by flow cytometry (AriaII, BD Biosciences), then cultured in complete RPMI 1640 medium.

### Plasmids

RNF185, cGAS, STING, TBK1, IRF3, AMFR, Trim32, Trim56, DDX41, DAI, IFI16 cDNAs were obtained by standard PCR techniques from thymus cDNA library and subsequently inserted into mammalian expression vectors as indicated. pCMV-Zeo-STING was kindly provided by Fanxiu Zhu (Florida State University, Tallahassee, USA). pET21b-PFO 85–1500 a.a. was a gift from Zhengfan Jiang (Peking University, Beijing, China). The reporter plasmids (IFNβ-luciferase and pTK-Renilla) have been described previously [61]. All point mutations were introduced by using a QuickChange XL site-directed mutagenesis method (Stratagene). All constructs were confirmed by sequencing.

### Antibodies and reagents

The rabbit polyclonal antibody against cGAS was from Cell Signaling Technology and Sigma-Aldrich. The polyclonal antibody against RNF185 was from Abcam. The antibodies against hemagglutinin (HA), Myc, GFP, and ubiquitin were purchased from Santa Cruz Biotechnology. Mouse monoclonal Flag antibody and β-actin antibody were obtained from Sigma-Aldrich. The TBK1 antibody was from Abcam. The IRF3 antibody was from Santa Cruz Biotechnology. Phospho-TBK1 and Phospho-IRF3 antibody was from Cell Signaling Technology. The antibodies against CoxIV, Calreticulin and Calnexin were from Abcam. Anti-Flag (M2)-agarose was from Sigma-Aldrich.

Herring testis (HT) DNA was from Sigma. Salmon sperm DNA was from TREVIGEN. Poly(I:C) was purchased from Invivogen. IFNα2a was from PBL Assay Science. cGAMP was obtained from InvivoGen. In some experiments, cGAMP was delivered into cultured cells by digitonin permeabilization method as previously described [62].

### RNA interference

Chemically synthesized 21-nucleotide siRNA duplexes were obtained from Invitrogen and Gene-Pharma, and transfected using Lipofectamine 2000 (Invitrogen) according to the manufacturer’s instructions. RNA oligonucleotides used in this study are as follows:

N.C.: 5-UUC UCC GAA CGU GUC ACG UTT-3;

*RNF185* #1: 5′- AAU CUU CCC UGG AAG CUU UTT-3′;

*RNF185* #2: 5′- GCC ACA GCA UUU AAC AUA ATT -3′.

### Reporter assays

Luciferase reporter assays were performed as described previously [63].

### Real-time RT-PCR

Total RNA was isolated from cultured cells using TRIzol reagent (Invitrogen) according to the manufacturer’s instructions, and then subjected to reverse transcription with PrimeScript RT Master Mix (Takara). The quantifications of gene transcripts were performed by real-time PCR using Power SYBR GREEN PCR MASTER MIX (ABI). GAPDH served as an internal control. PCR primers used to amplify the target genes were shown as follows:

*Gapdh*: sense (5′-GAA GGG CTC ATG ACC ACA GT-3′), antisense (5′-GGA TGC AGG GAT GAT GTT CT-3′); *Rnf185*: sense (5′-AGC AGA CTG GGA TTG TCT TG-3′); antisense (5′-CCA TTG CTG CTG CCA CTG GG -3′); *Ifnb*: sense (5′-AGA TCA ACC TCA CCT ACA GG-3′), antisense (5′-TCA GAA ACA CTG TCT GCT GG-3′); *Ifna4*: sense (5′-ACC CAC AGC CCA GAG AGT GAC C-3′), antisense (5′-AGG CCCT CTT GTT CCC GAG GT-3′); *Cxcl10*: sense (5′-CCT GCC CAC GTG TTG AGA T-3′), antisense (5′-TGA TGG TCT TAG ATT CCG GAT TC-3′); *GAPDH*: sense (5′-CGG AGT CAA CGG ATT TGG TC-3′), antisense (5′-GAC AAG CTT CCC GTT CTC AG-3′); *RNF185*: sense (5′-AGG ACC CCA GAG AGA AGA CC -3′), antisense (5′-CAA TTC CAA AAG ACA TCT GG-3′); *IFNB*: sense (5′-ATT GCC TCA AGG ACA GGA TG-3′), antisense (5′-GGC CTT CAG GTA ATG CAG AA-3′); *IFNA2*: sense (5′-CCT GAT GAA GGA GGA CTC CAT T-3′), antisense (5′-AAA AAG GTG AGC TGG CAT ACG-3′); *IFNA5*: sense (5′-TCC TCT GAT GAA TGT GGA CTC T-3′), antisense (5′-GTA CTA GTC AAT GAG AAT CAT TTC G-3′); *ISG15*: sense (5′-GAG AGG CAG CGA ACT CAT CT-3′), antisense (5′-CTT CAG CTC TGA CAC CGA CA-3′); *OASL-1*: sense (5′-CCA TCA CGG TCA CCA TTG TG-3′), antisense (5′-ACC GCA GGC CTT GAT CAG-3′).

### Immuno-precipitation analysis and immuno-blot analysis

Two-step immuno-precipitation and ubiquitination assays were performed as described previously [64]. For the first-round immunoprecipitation assay, cells were lysed by using Lysis buffer (50 mM Tris-Cl pH 7.4, 150 mM NaCl, 1% Triton X-100, 1 mM EDTA) supplemented with a protease inhibitor cocktail (Roche). Lysates were incubated with the anti-Flag (M2)-agarose for two hours. The immunoprecipitates were washed three times with the same buffer.

For the second-round immunoprecipitation assay, the immunoprecipitates were denatured by heating for 5 min in the Lysis buffer containing 1% SDS. The elutes were diluted by 10-fold with Lysis buffer followed by reimmunoprecipitating with the anti-Flag (M2)-agarose. After extensive wash, the immunoprecipitates were subjected to immunoblot analysis.

For denaturing immunoprecipitation, cells were lysed in 1% SDS buffer (50 mM Tris-HCl pH 7.5, 150 mM NaCl, 1% SDS, 10 mM DTT) and denatured by heating for thirty minutes. The lysates were centrifuged and diluted with Lysis buffer (50 mM Tris-HCl pH 7.5, 150 mM NaCl, 1 mM EDTA, 1% Triton X-100) until the concentration of SDS was decreased to 0.1%. The diluted lysates were immunoprecipitated with the indicated antibodies for four hours to overnight at 4°C before adding protein A/G agarose for two hours. After extensive wash, the immunoprecipitates were subjected to immunoblot analysis.

SDS-polyacrylamide gel electrophoresis (SDS-PAGE) and immunoblotting were performed as previously described[65].

### Native PAGE assay

Native gel electrophoresis for IRF3 dimerization was carried out as described previously [66].

### *In vitro* assay for cGAMP activity

The cGAMP activity assay was performed as described previously [21]. Briefly, L929 cells transfected with siRNAs were untreated or treated with HT-DNA for 6 hr and homogenized by douncing in the hypotonic buffer (10 mM Tris-HCl, pH 7.5, 10 mM KCl, 1.5 mM MgCl2, 1 mM DTT, 1mM PMSF) at 4°C. The homogenates were centrifuged at 100,000 RPM for 20 min at 4°C. After heated at 95°C for 5 min, the supernatant was centrifuged again at 12,000 RPM for 10 min to remove any precipitants. The heat-resistant lysates were mixed with fresh 10^6^ L929 cells in a 12.5 μl reaction containing 2 mM ATP, 1 U/μl of Benzonase and 2 ng/μl of PFO for 1.5 hr at 30°C. The cells were then subjected to the Native PAGE assay.

### *In vitro* ubiquitination assay

For the synthesis of polyUb chains, purified GST-RNF185 or mutants, His-Flag-cGAS, was incubated with E1 (50 nM), E2 (0.3 mM), ubiquitin (10 μM) (Boston Biochem) in a reaction buffer containing 50 mM Tris–HCl, pH 7.5, 5 mM MgCl_2_, 2 mM ATP, 2 mM DTT. The reaction was carried out at 37°C for 1 hr and then resolved by SDS-PAGE. Ubiquitinated products were detected by immunoblotting with indicated antibodies.

### *In vitro* cGAS enzymatic activity assay

RNF185 WT or RNF185 C39A catalyzed ubiquitinated cGAS or mutant was mixed with reaction buffer (20 mM HEPES, pH 7.5, 5 mM MgCl2, 2 mM ATP, 2 mM GTP) in the presence of Salmon sperm DNA. After incubation at 37°C for 45 min, the samples were centrifuged at 16,000 × g for 10 min. The product in the supernatant was separated from cGAS and DNA by passing through a 10kD ultrafiltration filter (Millipore). The samples were diluted by 5-fold and loaded onto a MonoQ ion exchange column (GE Healthcare) equilibrated with the running buffer (50 mM Tris-HCl pH 8.5) and eluted with a NaCl gradient of 0 to 0.5 M in the running buffer.

### Manipulation of viruses

HSV-1 and HSV-1-GFP were kindly provided by Dr. Wentao Qiao (Nankai University, Tianjin, China) and Dr. Chunfu Zheng (Suzhou University, Suzhou, China), respectively. HSV-1 was propagated and titered by plaque assays on Vero cells. SeV replication were measured as viral RNA expression using quantitative PCR with the following specific primers: sense (5′- GCT GCC GAC AAG GTG AGA GC -3′), antisense (5′- GCC CGC CAT GCC TCT CTC TA -3′). The MEF cells infected by HSV-1-GFP were quantified using a FACS Calibur (BD Biosciences) and the data was analyzed using Flowjo software (Tree Star).

### Measurement of cytokines

Concentrations of the cytokine in culture supernatants were measured by VeriKine Kit (PBL Assay Science) according to the manufacturer's instructions.

### Confocal microscopy

Confocal microscopy was performed as previously described [67]. Briefly, cells seeded onto glass coverslips were fixed with 4% paraformaldehyde in PBS for 20min, and then permeabilized with 0.1% Triton X-100 and blocked with 5% bovine serum albumin at room temperature. Then, the cells were incubated with the indicated primary antibodies followed by staining with fluorescent-conjugated secondary antibodies (Jackson Immuno-Research Laboratories). Nuclei were counterstained with DAPI (Sigma-Aldrich). For mitochondria staining, living cells were incubated with 300 nM Mito Tracker Red (Invitrogen) for 10 min at 37°C. Slides were mounted with fluorescent mounting medium (Dako). Imaging of the cells was carried out using Leica laser scanning confocal microscopy under a 64× oil objective.

### Subcellular fractionation

Mitochondria and ER membranes were purified on discontinuous sucrose gradients as previously described, with some modifications [24,68]. Briefly, MEF cells in ice-cold MTE buffer (0.27M mannitol, 10mM Tris-HCl, pH 7.4, 0.1mM EDTA) were lysed by using a dounce homogenizer. Lysed cells were centrifuged at 700g for 10 min, and the supernatant was collected. The supernatant was then centrifugated at 15,000g for 10 min to obtain the crude Mitochondria fraction, and post mitochondrial supernatant was used for purification of ER fractions. The crude mitochondria pellet was resuspended in MTE buffer, and was layered on top of the discontinuous sucrose gradients (1.0M and 1.7M sucrose in 10mM Tris-HCl, pH 7.5) and centrifugated at 40,000g for 22 min. Mitochondria fraction was collected and pelleted by centrifugation at 15,000g for 10 min. Purified mitochondria were resuspended in PBS and prepared for western blot analysis. To isolate ER fractions, post-mitochondrial supernatant was layered on discontinuous sucrose gradients (1.3 M, 1.5M and 2.0M sucrose in 10mM Tris-HCl, pH 7.6) and centrifugated at 100,000g for 70 min. The ER fraction at the interface between the supernatant and the 1.3M sucrose was collected, and pelleted by centrifugation at 100,000g for 45 min. The purified ER membranes were resuspended in PBS and prepared for western blot analysis.

### Statistics

Student’s t test was used for the statistical analysis of two independent treatments. The difference in RNF185 mRNA level, ISG15 mRNA level or OASL-1 mRNA level between subject groups was analyzed by Mann-Whitney test. For all tests, a P value of < 0.05 was considered statistically significant.

## Supporting information

S1 Fig RNF185 is homologous to RNF5. (A) Amino acid sequence alignment by the DNAMAN software was shown for RNF185 (*Homo sapiens*), RNF185 (*Mus musculus*), RNF5 (*Homo sapiens*), and RNF5 (*Mus musculus*).
(TIF)

S2 Fig Increased IFN production does not lead to upregulation of RNF185 mRNA expression. (A) The indicated siRNAs were transfected into L929 cells. Forty-eight hours later, cells were stimulated with HT-DNA or poly(I:C), followed by assessing the induction of *Ifnb*, *Ifna4* and *Cxcl10* mRNAs using quantitative PCR. (B) PBMCs were stimulated with IFNα 2a in different dose gradients for 16h, followed by assessing the induction of *RNF185*, *ISG15* and *OASL-1* mRNAs using quantitative PCR. (C) PBMCs were stimulated with IFNα 2a (1×10^3^ U/ml) in early time points (2h and 4h), followed by assessing the induction of *RNF185*, *ISG15* and *OASL-1* mRNAs using quantitative PCR. (D) PBMCs were stimulated with the 25% serum or 50% serum from healthy donors and SLE patients for 12h. Induction of *RNF185*, *ISG15* and *OASL-1* mRNAs was measured by quantitative PCR. (E) PBMCs were stimulated with the 25% serum or 50% serum from healthy donors and SLE patients in early time points (2h and 4h). Induction of *RNF185*, *ISG15* and *OASL-1* mRNAs was measured by quantitative PCR. Data from A-E are presented as means ± S.D. from three independent experiments. *, *P* < 0.05; **, *P* < 0.01. n.s., not significant.
(TIF)

S3 Fig Silencing of *Rnf185* inhibits the HSV-1 induced expression of IRF3-responsive genes in RAW264.7 cells and BMDMs. (A and B) The indicated siRNAs were transfected into RAW264.7 cells. Induction of *Ifnb*, *Ifna4* and *Cxcl10* mRNAs was measured by quantitative PCR after HSV-1 (MOI = 5) invasion (A) or SeV infection (B) for 6h. (C) BMDMs were transfected with the negative control (N.C.) or *Rnf185* siRNAs. Cell lysates were immunoblotted with the indicated antibodies. (D and E) The indicated siRNAs were transfected into BMDMs. Induction of *Ifnb*, *Ifna4* and *Cxcl10* mRNAs was measured by quantitative PCR after HSV-1 (MOI = 5) invasion (D) or SeV infection (E) for 6h. (F) BMDMs transfected with the indicated siRNAs were infected with HSV-1 (MOI = 5) or SeV (50 HAU/ml) for 36h. The titer of HSV-1(left panel) was determined by standard plaque assay, and SeV (right panel) replication was determined by detection of SeV RNA by quantitative PCR. Data from A, B, D-F are presented as means ± S.D. from three independent experiments. **, *P* < 0.01. n.s., not significant.
(TIF)

S4 Fig Silencing of RNF185 attenuates exogenous DNA-induced TBK1 phosphorylation. (A and B) The indicated siRNAs were transfected into L929 cells. Forty-eight hours after transfection, cells were treated with HT-DNA (A) or poly(I:C) (B) for the indicated time periods, and cell extracts were analyzed for TBK1 phosphorylation. (C) L929/cGAS cells were transfected with the negative control (N.C.) or *Rnf185* siRNA. 48h after transfection, cells were treated with or without HT-DNA. Induction of *Ifnb* mRNA was measured by quantitative PCR. Data are presented as means ± S.D. from three independent experiments. **, *P* < 0.01. (D) L929/cGAS cells were transfected with the negative control (N.C.) or *Rnf185* siRNA. 48h after transfection, cells were treated with or without HT-DNA, and cell extracts were analyzed for the phosphorylation of TBK1 and IRF3.
(TIF)

S5 Fig RNF185 catalyzes K27-linked poly-ubiquitination of cGAS. (A) Quantification of colocalization of RNF185 and cGAS in Fig 3F based on the Pearson’s correlation coefficient (a perfect linear correlation is +1) was determined by the Volocity software. (B) HEK293T cells were transfected with Flag-tagged cGAS and Myc-tagged RNF185 along with Ub or its mutants. Cell lysates were subjected to a two-step immunoprecipitation, and then immunoblotted with the indicated antibodies. (C) L929/cGAS WT cells and L929/cGAS K173/384R cells were transfected with or without HT-DNA. Induction of *Ifnb* and *Cxcl10* mRNAs was measured by quantitative PCR. (D) L929/cGAS WT cells and L929/cGAS K173/384R cells were transfected with or without HT-DNA, and cell extracts were analyzed for the phosphorylation of TBK1 and IRF3. Data from A and C are presented as means ± S.D. from three independent experiments. **, *P* < 0.01.
(TIF)

S6 Fig Identification of RNF185 as a new regulator of the innate immune response to cytosolic DNA by an unbiased RNAi-based screening approach. The indicated individual siRNA oligos were transfected into L929 cells. Induction of *Ifnb* mRNA was measured by quantitative PCR after HSV-1 (MOI = 0.5) infection for 6h. The proteins with at least a 2-fold decrease compared to the control were defined as the positive candidates (shown in red): RNF185, RNF45 (a.k.a. AMFR), RNF128.
(TIF)
