# Supplementary figures and images for: The E3 ubiquitin ligase RNF185 facilitates the cGAS-mediated innate immune response
Source: PLoS Pathog. 2017 Mar 8;13(3):e1006264. doi: 10.1371/journal.ppat.1006264 (PMC5358892; doi:10.1371/journal.ppat.1006264)

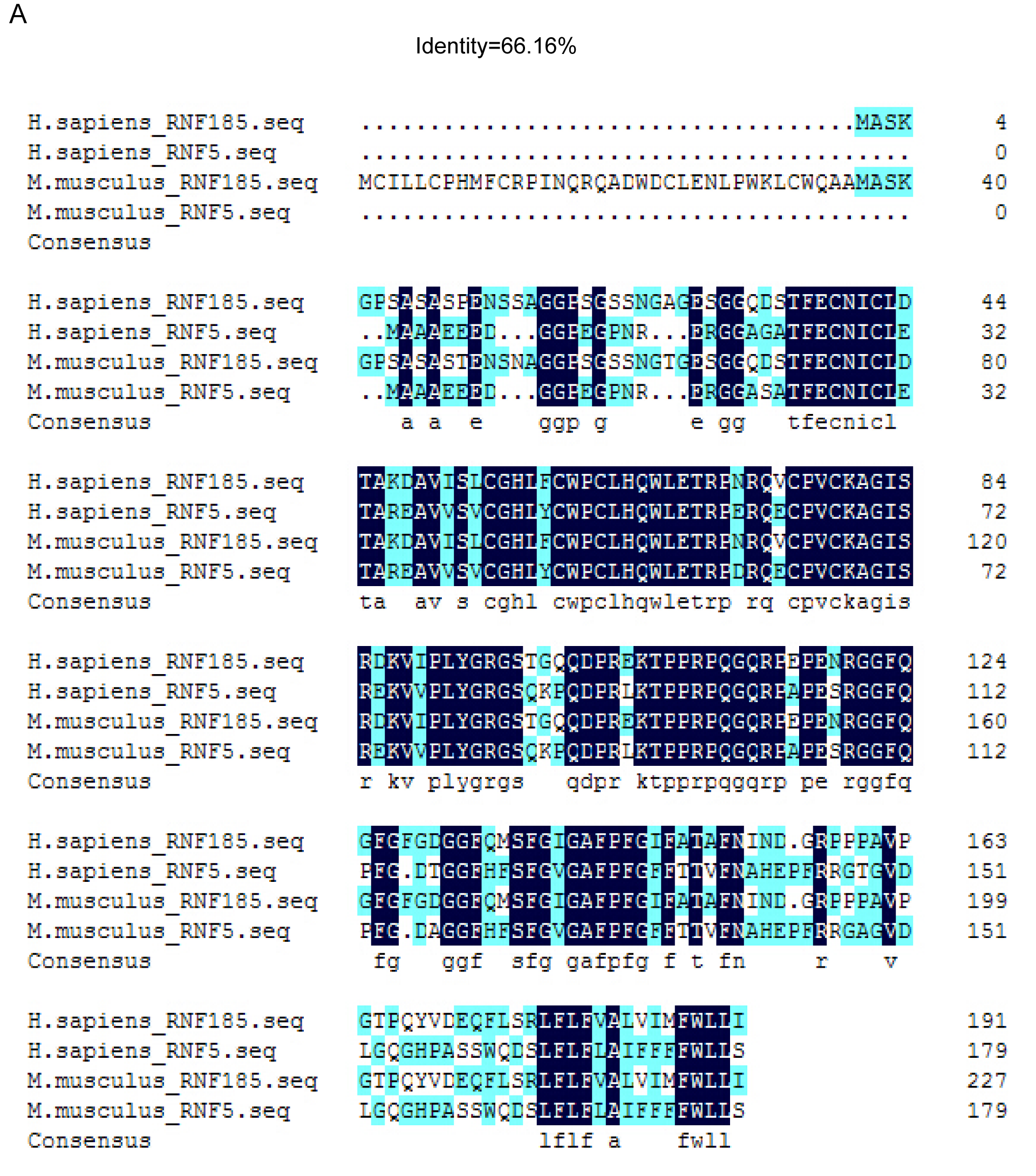

Supplement: S1 Fig — (A) Amino acid sequence alignment by the DNAMAN software was shown for RNF185 (Homo sapiens), RNF185 (Mus musculus), RNF5 (Homo sapiens), and RNF5 (Mus musculus). (TIF) [file ppat.1006264.s001.tif]

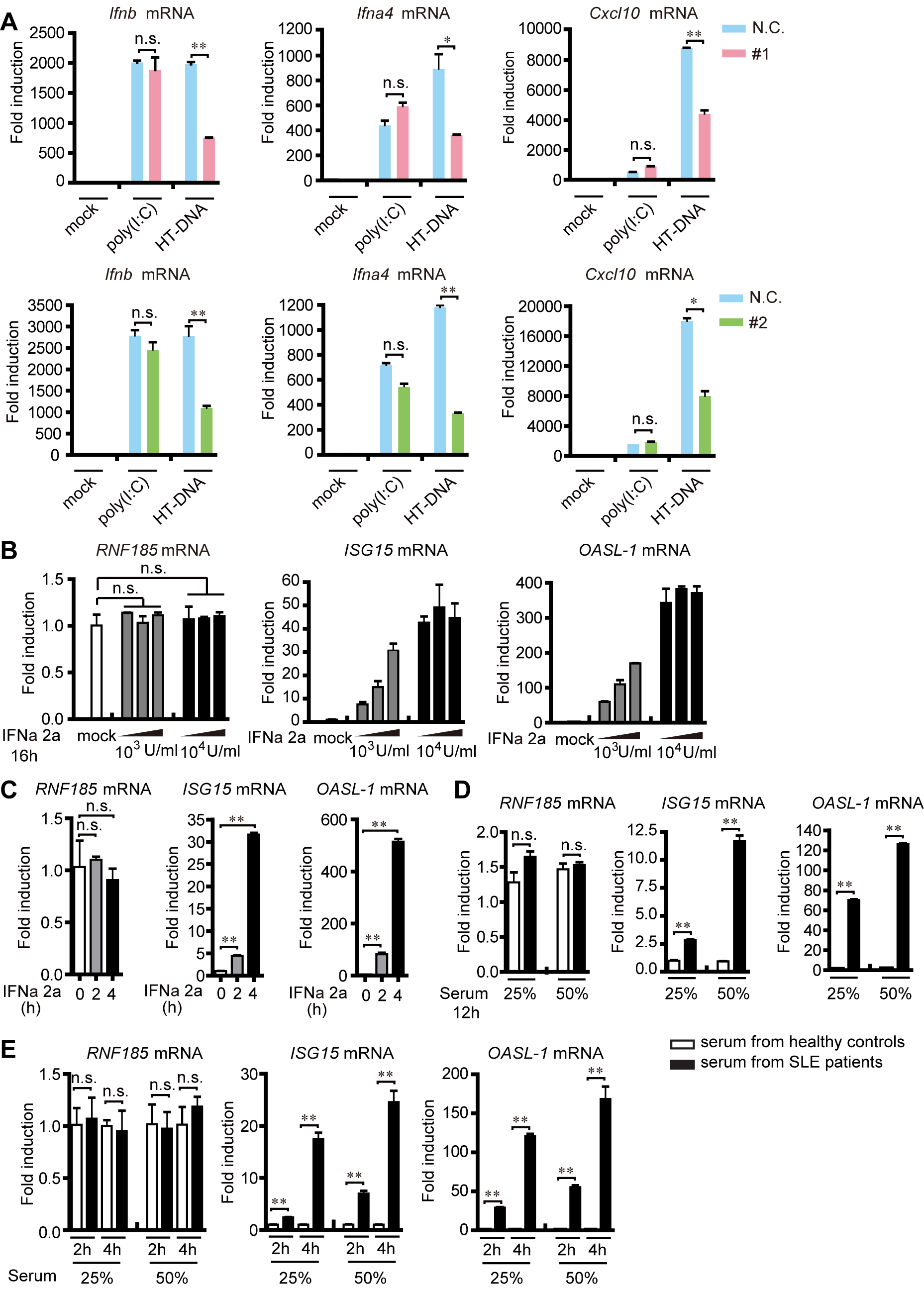

Supplement: S2 Fig — (A) The indicated siRNAs were transfected into L929 cells. Forty-eight hours later, cells were stimulated with HT-DNA or poly(I:C), followed by assessing the induction of Ifnb, Ifna4 and Cxcl10 mRNAs using quantitative PCR. (B) PBMCs were stimulated with IFNα 2a in different dose gradients for 16h, followed by assessing the induction of RNF185, ISG15 and OASL-1 mRNAs using quantitative PCR. (C) PBMCs were stimulated with IFNα 2a (1×10^3^ U/ml) in early time points (2h and 4h), followed by assessing the induction of RNF185, ISG15 and OASL-1 mRNAs using quantitative PCR. (D) PBMCs were stimulated with the 25% serum or 50% serum from healthy donors and SLE patients for 12h. Induction of RNF185, ISG15 and OASL-1 mRNAs was measured by quantitative PCR. (E) PBMCs were stimulated with the 25% serum or 50% serum from healthy donors and SLE patients in early time points (2h and 4h). Induction of RNF185, ISG15 and OASL-1 mRNAs was measured by quantitative PCR. Data from A-E are presented as means ± S.D. from three independent experiments. *, P < 0.05; **, P < 0.01. n.s., not significant. (TIF) [file ppat.1006264.s002.tif]

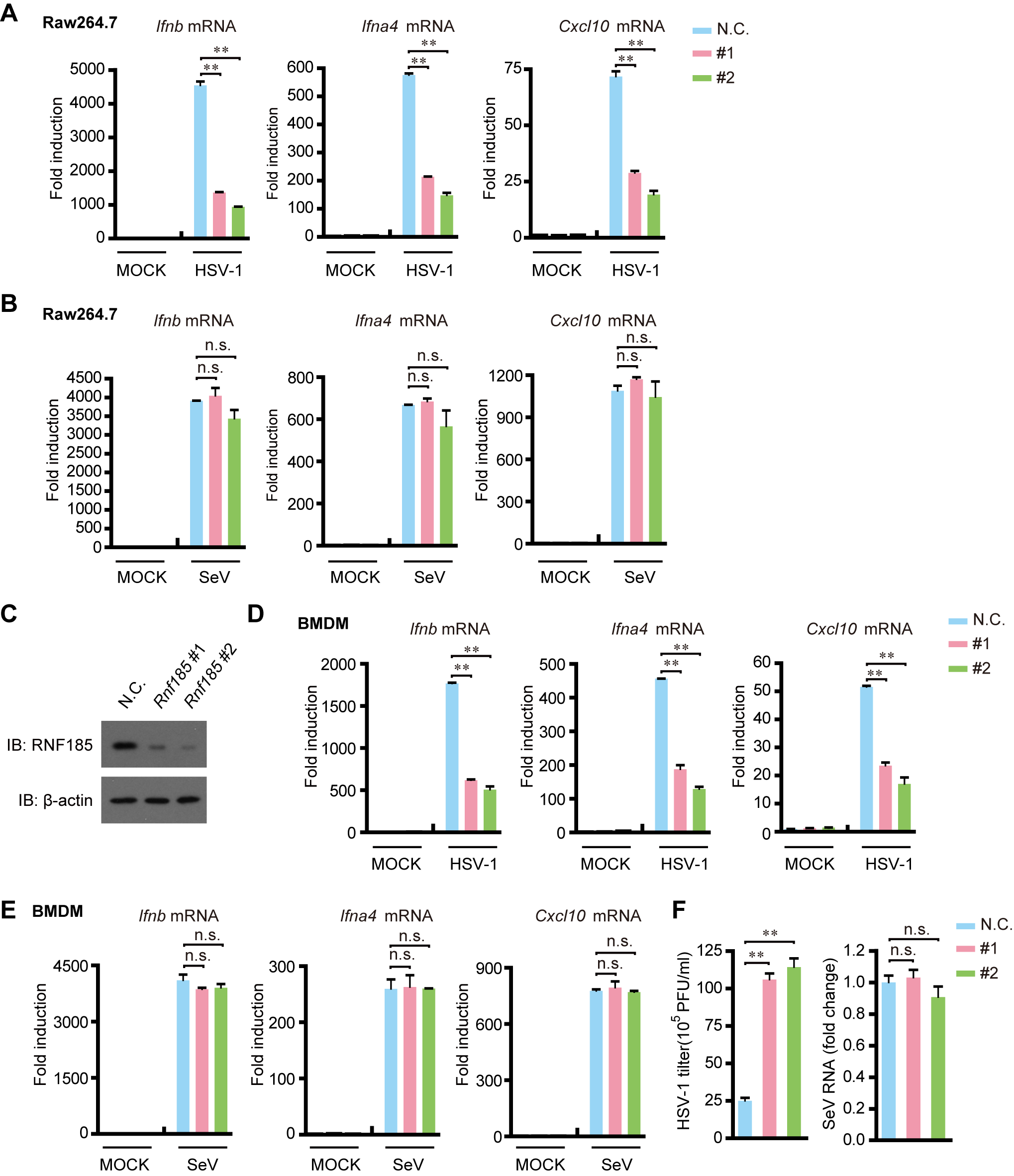

Supplement: S3 Fig — (A and B) The indicated siRNAs were transfected into RAW264.7 cells. Induction of Ifnb, Ifna4 and Cxcl10 mRNAs was measured by quantitative PCR after HSV-1 (MOI = 5) invasion (A) or SeV infection (B) for 6h. (C) BMDMs were transfected with the negative control (N.C.) or Rnf185 siRNAs. Cell lysates were immunoblotted with the indicated antibodies. (D and E) The indicated siRNAs were transfected into BMDMs. Induction of Ifnb, Ifna4 and Cxcl10 mRNAs was measured by quantitative PCR after HSV-1 (MOI = 5) invasion (D) or SeV infection (E) for 6h. (F) BMDMs transfected with the indicated siRNAs were infected with HSV-1 (MOI = 5) or SeV (50 HAU/ml) for 36h. The titer of HSV-1(left panel) was determined by standard plaque assay, and SeV (right panel) replication was determined by detection of SeV RNA by quantitative PCR. Data from A, B, D-F are presented as means ± S.D. from three independent experiments. **, P < 0.01. n.s., not significant. (TIF) [file ppat.1006264.s003.tif]

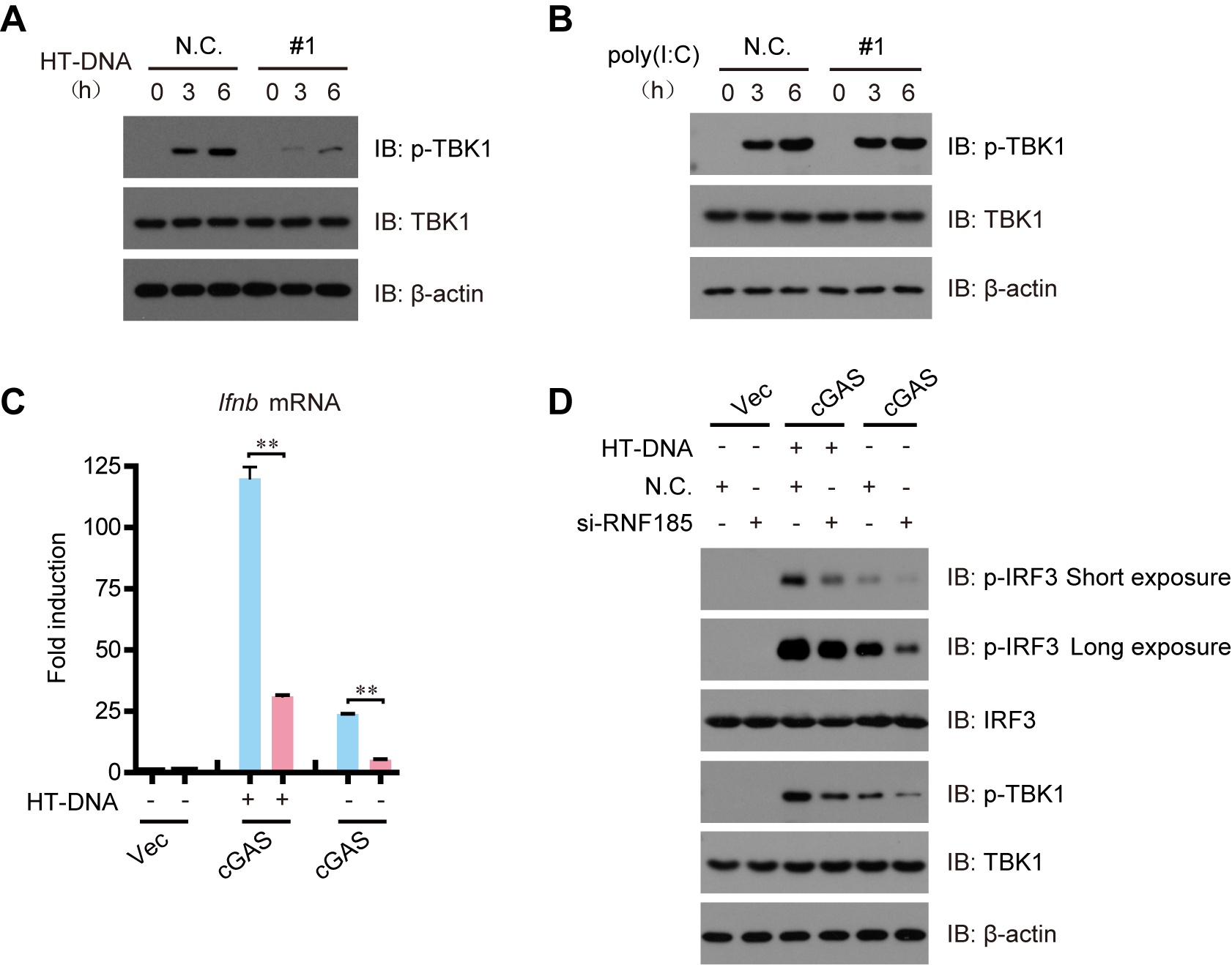

Supplement: S4 Fig — (A and B) The indicated siRNAs were transfected into L929 cells. Forty-eight hours after transfection, cells were treated with HT-DNA (A) or poly(I:C) (B) for the indicated time periods, and cell extracts were analyzed for TBK1 phosphorylation. (C) L929/cGAS cells were transfected with the negative control (N.C.) or Rnf185 siRNA. 48h after transfection, cells were treated with or without HT-DNA. Induction of Ifnb mRNA was measured by quantitative PCR. Data are presented as means ± S.D. from three independent experiments. **, P < 0.01. (D) L929/cGAS cells were transfected with the negative control (N.C.) or Rnf185 siRNA. 48h after transfection, cells were treated with or without HT-DNA, and cell extracts were analyzed for the phosphorylation of TBK1 and IRF3. (TIF) [file ppat.1006264.s004.tif]

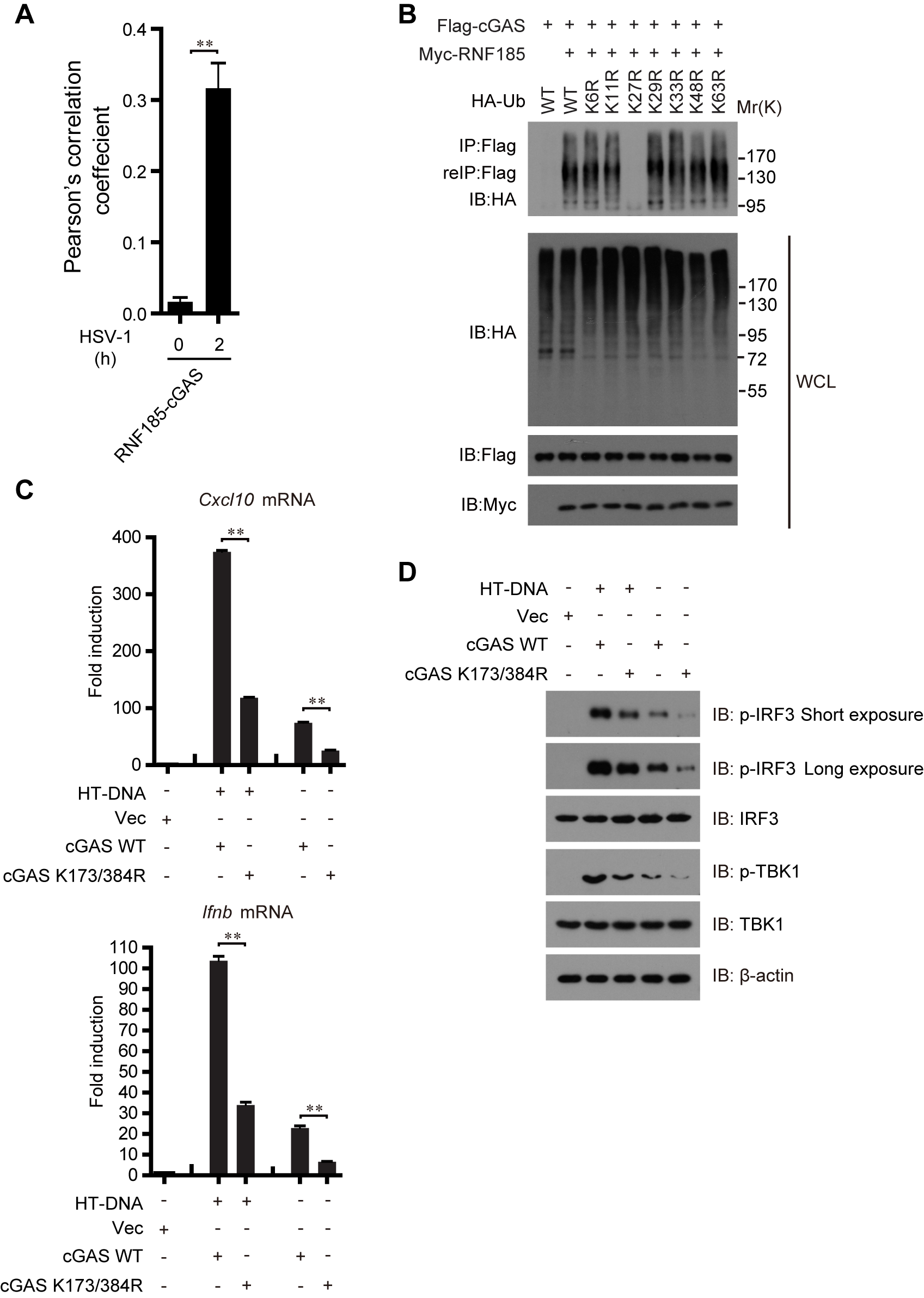

Supplement: S5 Fig — (A) Quantification of colocalization of RNF185 and cGAS in Fig 3F based on the Pearson’s correlation coefficient (a perfect linear correlation is +1) was determined by the Volocity software. (B) HEK293T cells were transfected with Flag-tagged cGAS and Myc-tagged RNF185 along with Ub or its mutants. Cell lysates were subjected to a two-step immunoprecipitation, and then immunoblotted with the indicated antibodies. (C) L929/cGAS WT cells and L929/cGAS K173/384R cells were transfected with or without HT-DNA. Induction of Ifnb and Cxcl10 mRNAs was measured by quantitative PCR. (D) L929/cGAS WT cells and L929/cGAS K173/384R cells were transfected with or without HT-DNA, and cell extracts were analyzed for the phosphorylation of TBK1 and IRF3. Data from A and C are presented as means ± S.D. from three independent experiments. **, P < 0.01. (TIF) [file ppat.1006264.s005.tif]

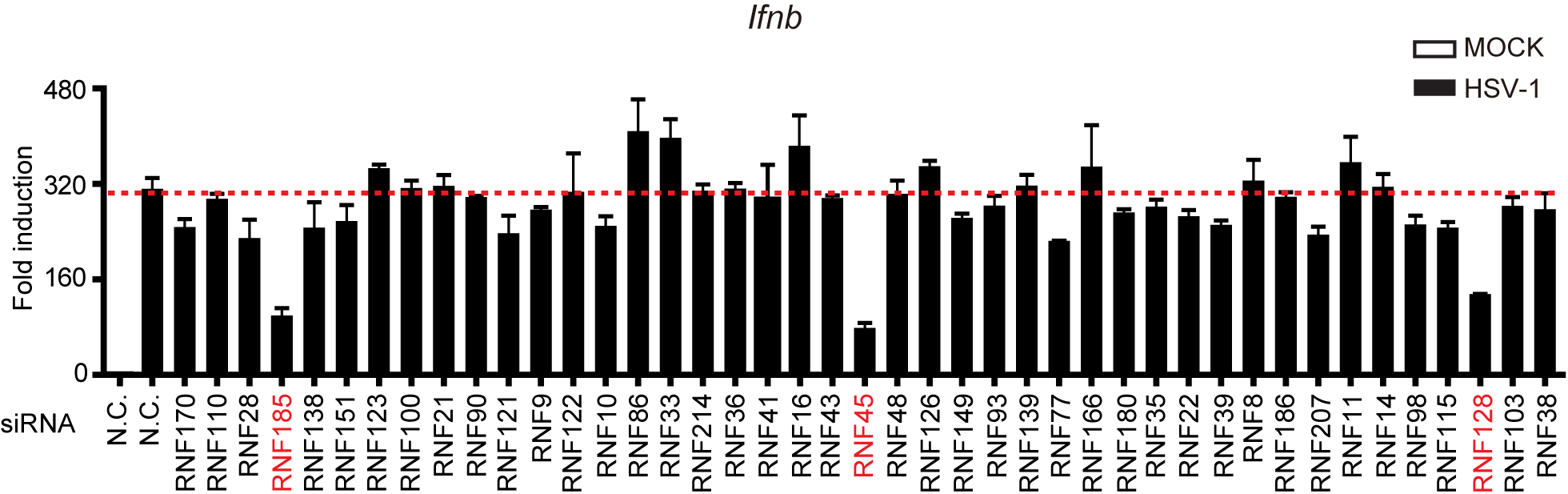

Supplement: S6 Fig — The indicated individual siRNA oligos were transfected into L929 cells. Induction of Ifnb mRNA was measured by quantitative PCR after HSV-1 (MOI = 0.5) infection for 6h. The proteins with at least a 2-fold decrease compared to the control were defined as the positive candidates (shown in red): RNF185, RNF45 (a.k.a. AMFR), RNF128. (TIF) [file ppat.1006264.s006.tif]
